# The TOBY Study. Whole body hypothermia for the treatment of perinatal asphyxial encephalopathy: A randomised controlled trial

**DOI:** 10.1186/1471-2431-8-17

**Published:** 2008-04-30

**Authors:** Dennis Azzopardi, Peter Brocklehurst, David Edwards, Henry Halliday, Malcolm Levene, Marianne Thoresen, Andrew Whitelaw, The TOBY Study Group

**Affiliations:** 1 Division of Clinical Sciences, Faculty of Medicine, Imperial College London, UK; 2 National Perinatal Epidemiology Unit, University of Oxford, UK; 3 Royal Maternity Hospital Belfast, Northern Ireland, UK; 4 Academic Unit of Paediatrics, Obstetrics & Gynaecology Clarendon Wing Leeds General Infirmary, Leeds, UK; 5 St. Michaels Hospital, Southwell Street, Bristol, UK University of Bristol Medical School Unit, Southmead Hospital, Bristol, UK

## Abstract

**Background:**

A hypoxic-ischaemic insult occurring around the time of birth may result in an encephalopathic state characterised by the need for resuscitation at birth, neurological depression, seizures and electroencephalographic abnormalities. There is an increasing risk of death or neurodevelopmental abnormalities with more severe encephalopathy. Current management consists of maintaining physiological parameters within the normal range and treating seizures with anticonvulsants.

Studies in adult and newborn animals have shown that a reduction of body temperature of 3–4°C after cerebral insults is associated with improved histological and behavioural outcome. Pilot studies in infants with encephalopathy of head cooling combined with mild whole body hypothermia and of moderate whole body cooling to 33.5°C have been reported. No complications were noted but the group sizes were too small to evaluate benefit.

**Methods/Design:**

TOBY is a multi-centre, prospective, randomised study of term infants after perinatal asphyxia comparing those allocated to "intensive care plus total body cooling for 72 hours" with those allocated to "intensive care without cooling".

Full-term infants will be randomised within 6 hours of birth to either a control group with the rectal temperature kept at 37 +/- 0.2°C or to whole body cooling, with rectal temperature kept at 33–34°C for 72 hours. Term infants showing signs of moderate or severe encephalopathy +/- seizures have their eligibility confirmed by cerebral function monitoring. Outcomes will be assessed at 18 months of age using neurological and neurodevelopmental testing methods.

**Sample size:**

At least 236 infants would be needed to demonstrate a 30% reduction in the relative risk of mortality or serious disability at 18 months.

Recruitment was ahead of target by seven months and approvals were obtained allowing recruitment to continue to the end of the planned recruitment phase. 325 infants were recruited.

**Primary outcome:**

Combined rate of mortality and severe neurodevelopmental impairment in survivors at 18 months of age. Neurodevelopmental impairment will be defined as any of:

• Bayley mental developmental scale score less than 70

• Gross Motor Function Classification System Levels III – V

• Bilateral cortical visual impairments

**Trial Registration:**

Current Controlled Trials ISRCTN89547571

## Background

This is a multicentre prospective randomised controlled trial to determine whether a reduction of body temperature by 3–4°C following perinatal asphyxia improves survival without neurodevelopmental disability. Full term infants will be randomised within 6 hours of birth to either a control group with the rectal temperature kept at 37 ± 0.2°C or to whole body cooling with the rectal temperature kept at 33.5 ± 0.5°C for 72 hours followed by slow rewarming. The outcome will be assessed at 18 months of age by survival and neurological and neurodevelopmental testing.

### Hypothesis

Prolonged whole body cooling in term infants with perinatal asphyxial encephalopathy reduces death and severe neurodevelopmental disability.

### Perinatal asphyxial encephalopathy

A hypoxic-ischaemic insult occurring around the time of birth may result in an encephalopathic state characterised by the need for resuscitation at birth, neurological depression, seizures and electroencephalographic abnormalities. There is an increasing risk of death or neurodevelopmental abnormalities with more severe encephalopathy [1].

### Pathogenesis of perinatal asphyxial encephalopathy

Experimental studies [2-4] have shown that a severe perinatal hypoxic-ischaemic insult (HII) results in an evolving process of adverse biochemical events that include increased levels of neurotransmitters, excessive production of free radicals, increased intracellular calcium, and stimulation of inflammatory mediators and messengers that initiate apoptotic cell death.

Alterations in cerebral energy metabolism can be observed during and following HII. During HII cerebral metabolism is impaired and cerebral high-energy phosphate levels fall precipitously. Following termination of the insult, cerebral energy metabolism initially recovers but may then deteriorate 6–24 hours later. This secondary energy failure is not contingent on impaired substrate supply and its severity is related to survival, head growth and neurodevelopmental outcome at 1 and 4 years [5-7]. Although the secondary phase of impaired cerebral energy metabolism resolves after about 72 hours, a persistent disturbance may be detected for several months [8,9].

### Current management of perinatal asphyxial encephalopathy

No specific treatments are presently available. Current management consists of maintaining physiological parameters within the normal range and treating seizures with anticonvulsants.

### Experimental treatments

Several pharmacological agents have shown benefit in small animal models of hypoxic ischaemic injury [10-12]. However, these observations may have limited relevance to the clinical situation, where the onset and severity of the insult are poorly defined and prophylactic treatment is not feasible. This may explain the lack of benefit of agents such as calcium channel antagonists and magnesium in term infants [13].

Recently, pilot studies of hypothermia, phenobarbitone and allopurinol have been reported. Although the severity of seizures was not influenced, very early treatment with high dose phenobarbitone reduced handicap at 3 years [14]. High dose allopurinol improved electroencephalographic (EEG) activity and reduced free radical formation in 22 neonates, but long-term outcomes were not assessed [15]. However, previous studies have not consistently shown benefit [16].

### Experimental treatment with hypothermia

Studies in adult and newborn animals have shown that a reduction of body temperature of 3–4°C after cerebral insults is associated with improved histological and behavioral outcome [17-19]. Several mechanisms may help explain these benefits: the increases in extracellular glutamate and free radical and nitric oxide synthesis are suppressed [2], cerebral energy phosphates are preserved and cerebral alkalosis and lactate reduced [3,20]. Mild hypothermia may also suppress the activation of microglia [21-23]. These changes may preserve cerebral energy metabolism, reduce cytotoxic cerebral oedema and intracranial pressure and inhibit apoptosis.

### Timing of hypothermia treatment

Factors that may critically affect the therapeutic response to hypothermia are the interval between the insult and initiation of hypothermia, the severity of the insult and the duration and depth of hypothermia. Most studies indicate that the maximum benefit occurs when treatment is started within six hours of the insult [17,24], although limited neuroprotection has been observed when hypothermia is started as late as 12 hours. Therefore, hypothermic treatment following perinatal asphyxia will need to be started as soon as practically possible. Limited benefit only might be expected if treatment is delayed beyond six hours after birth.

### Duration of treatment with hypothermia

The duration of treatment with hypothermia has varied greatly in experimental studies. Consistently beneficial effects have been observed when moderate hypothermia was maintained for 12–24 hours, but it has been suggested that treatment up to 72 hours may be required if the interval before inducing hypothermia is prolonged or milder degrees of hypothermia are employed [25]. A rebound rise in epileptiform activity in fetal lambs [25] and in intracranial pressure in adults with stroke [26] have been reported when hypothermia was discontinued before 72 hours.

### Clinical trials of treatment with hypothermia

Preliminary studies in adults of whole body hypothermia following head injury [27], cardiac arrest [28-30] and stroke [26] have suggested benefit. In 2001 a systematic review of treatment of head injury with whole body hypothermia failed to demonstrate benefit but this may have been due to inconsistencies in clinical management [31,32].

In neonates, hypothermia was first reported as therapy for resuscitation, before modern resuscitation techniques were introduced. Pilot studies in infants with encephalopathy of head cooling combined with mild whole body hypothermia and of moderate whole body cooling to 33.5°C have been reported [33-35]. No complications were noted but the group sizes were too small to evaluate benefit.

### Potential adverse effects of hypothermia

The risk of adverse effects of hypothermia is a critical issue, given the poor outcome conventionally associated with hypothermia [36,37]. Impaired cardiac function, disordered coagulation and increased sepsis have been reported with profound hypothermia. Thrombocytopenia, hypokalaemia and increased sepsis have been noted in studies of hypothermia to 33°C in adults, but these were not associated with an adverse outcome [26]. The metabolic rate is reduced during hypothermia and this results in bradycardia and prolonged PR and QT intervals. However, significant arrhythmia has only been reported in adults with head injury when the rectal temperature is less than 32°C [38].

There are few data on the safety of mild or moderate hypothermia in newborn infants since previous studies employed hypothermia for brief periods in the treatment of failed resuscitation [39-42]. These early studies appeared to demonstrate improved outcomes without side effects but few controls were included.

No clinically significant adverse effects due to cooling were observed in the recent pilot studies of whole body cooling following encephalopathy [34,35]. As expected, the heart rate reduced to below 100 bpm with cooling but this was not accompanied by arrhythmia, and the blood pressure and perfusion were maintained.

### Selective or whole body cooling

Although some experimental evidence suggests that selective head cooling may be effective [25,43], it is uncertain whether head cooling alone effectively lowers deep brain temperature. In anaesthetized piglets, which have a much smaller head than the human newborn infant, it was possible to cool the basal ganglia 5°C more than rectal temperature [44]. In adults, a steep temperature gradient has been observed between the surface of the head and deep brain structures and the deep brain temperature remains very close to core temperature even when extreme cold is applied to the surface of the head [45]. The possibility of temperature gradients within the brain and the importance of damage to deep structures in causing severe neurodevelopmental impairment [46] and the lack of precise, non-invasive methods for measuring regional brain temperature pose major difficulties with selective head cooling.

### Pilot studies of whole body hypothermia in neonates

Pilot studies of prolonged whole body cooling in infants with perinatal asphyxial encephalopathy have been carried out [34,35]. Infants were selected on staged criteria based on the clinical condition at birth and abnormal amplitude integrated EEG (aEEG). These criteria have previously been shown to have > 70% positive predictive value for death or severe neurodevelopmental disability [47]. Selection was completed, written informed consent was obtained and hypothermic treatment started within six hours of birth, including the infants born outside the study centres. Rectal temperature was maintained at 33–34°C for 48–72 hours.

An increase in blood pressure and a reduction in heart rate with cooling were noted, but these were not clinically significant, unless there was an inadvertent rapid change in body temperature [34]. A mild hypokalaemia was commonly observed. No disturbance in coagulation or viscosity attributable to cooling was seen.

Magnetic resonance imaging (MRI) was performed repeatedly. Sinus thrombosis was observed in two infants and one infant developed delayed haemorrhagic cerebral infarction. These three infants died after intensive care was withdrawn according to parental wishes when the clinical, EEG and MRI findings indicated severe, irreversible cerebral injury.

### Clinical trials of hypothermia in neonates

Since the TOBY trial was started in 2002, three other trials of hypothermia for the management of neonatal encephalopathy (NE) (one using selective head cooling and two using whole body cooling), have been published [73-75]. The results of these trials are summarised here with a reminder of the assumptions that were made when calculating the required sample size of the TOBY study:

### Table 1: Summary of published hypothermia trials, showing effect size

**Table 1:** Summary of published hypothermia trials, showing effect size

**Name of trial**	**Number in trial**	**Primary outcome and main secondary outcome**	**Effect size [assumed for TOBY]**
Cool cap trial	218	Death or severe impairment at 18 months	RR 0.82 95%CI 0.66–1.02
NICHD Trial	208	Death or any impairment at 18 months	RR 0.73 95%CI 0.56–0.95
Eicher	65	Death or severe impairment at 12 months	RR 0.62 95%CI 0.41–0.92
TOBY	236	Death or severe impairment at 18 months	[RR 0.70]

Despite the promising results which are beginning to emerge from the completed studies, none of the completed or on-going studies (including TOBY) are large enough to allow assessment of moderate differences after 18 months of age. There is increasing concern that therapeutic hypothermia may be introduced into clinical practice without any knowledge of whether these apparent initial benefits are maintained in the longer term, such as at 6 years of age.

There is broad support for continuing to recruit to the on going trials: a meeting convened by the NIH in Washington to assess future studies of treatment with hypothermia in neonates concluded that:

• "Hypothermic treatment for birth asphyxia is not a standard of care but an investigational treatment with promise of benefit;"

• "There was agreement that current randomised controlled trials should be completed and a high priority was put upon this essential data and continuation of the ongoing trials was believed to be essential;"

• "It was agreed that long term follow-up data were essential and strongly advocated."

### Early identification of infants at risk of perinatal asphyxial encephalopathy Clinical features

Perinatal asphyxial encephalopathy is reported to occur in between one and two infants per thousand deliveries, and much more frequently in countries without adequate obstetric facilities. Early identification by clinical features of infants at risk of developing encephalopathy is difficult. Fetal heart rate patterns are not very helpful [48,49] and neither the Apgar scores recorded immediately after birth [50,51] nor umbilical cord blood gas analysis [52,53] are good predictors of the likelihood or severity of post asphyxial encephalopathy. However, a continuing need for resuscitation and acidaemia with a cord blood pH < 7.0, are considered essential features of perinatal asphyxia.

### Neuroimaging

Abnormal appearances can be observed on cranial ultrasonography, computerized tomography [54] and MRI [55]. However, these abnormalities may not be apparent during the first 24 – 48 hours after birth. Therefore, these techniques may be of use as prognostic indicators but not for early selection of infants for neuroprotective therapies.

### Amplitude integrated EEG (aEEG)

Standard electroencephalography (EEG) and measurement of evoked potentials may provide objective evidence for abnormal cerebral function following perinatal asphyxia [56,57]. However, these techniques are difficult to apply very soon after birth.

Continuous aEEG during the first six hours after birth provides a more simple objective measure for the presence of encephalopathy [57,58]. The aEEG records a single channel EEG from 2 biparietal electrodes. The filtered signal, containing the main EEG frequencies, is rectified, smoothed and amplitude integrated before it is written out, at the bedside on slow-speed paper (6 cm/hour). It is easier to interpret the aEEG than the conventional EEG, although it gives less information on the underlying type of activity.

There is a good correlation between early continuous amplitude monitoring and the conventional EEG [47,59]. The background aEEG pattern after perinatal asphyxia is reported to accurately predict the outcome in 91% of infants with low Apgar scores. This represents a positive predictive value for adverse outcome of approximately 80% [47,57]. When recordings were performed during the first 3 postnatal hours, the positive predictive value was 75%. However, only 6 of 37 infants showed a change in aEEG pattern between 3 and 6 hours after birth [53]. Most of the infants showing a change from the initial aEEG showed an improvement.

Only infants with a moderately or severely abnormal aEEG defined using a semiquantitative classification system [47] will be recruited to TOBY. Such patients show aEEG background activity with the upper margin below 10 microvolts and/or a lower margin below 5 microvolts. There was an excellent correlation (Kappa 0.87) between the scores of two paediatric registrars who had received minimal training in the use of these criteria and an expert [47].

### Selection criteria for study entry

The use of multiple clinical risk factors as entry criteria would ensure that most babies recruited have perinatal asphyxia. However, the sensitivity would be low, so most of the babies with perinatal asphyxia would be missed [52,60]. A more effective approach is to combine an initial screening test such as severe acidosis or low Apgar score at birth (< 5 at 5 or 10 min), with abnormal neurological signs and abnormal amplitude integrated EEG recordings during the first few hours of life.

### Duration of Follow-up

The minimum duration of follow-up that is required for accurately diagnosing neuromotor, neurosensory and cognitive disability is 18 months. Long-term follow-up to at least 6 years of age will also be desirable, as a secondary study, for detailed assessment of intellectual function.

There is now strong experimental evidence in studies of adult and immature animals that the short term histological and behavioural neuroprotection seen with prolonged post-insult hypothermia [61,62] leads to long lasting neuroprotection associated with corresponding functional improvement [17,63-65]. Such long lasting functional neuroprotection has been reported despite up to 6 hours delay in initiating cooling in adult animal models [64-66].

Clinical data from follow-up of adults and children subjected to deep hypothermia during surgery have found no evidence for delayed adverse effects [67,68].

## Methods/Design

This will be a multi-centre, prospective, randomised study of term infants after perinatal asphyxia comparing those allocated to "intensive care plus total body cooling for 72 hours" with those allocated to "intensive care without cooling". Clinical staff will be unblinded because they must know the rectal temperature of the infants in order to adjust the cooling and heating appropriately. The primary outcome measure (severe neurodevelopmental disability) will be assessed blind to allocation.

### Inclusion criteria

The infant will be assessed sequentially by criteria A, B and C listed below:

**A. **Infants ≥ 36 completed weeks gestation admitted to the NICU with at least one of the following:

• Apgar score of ≤ 5 at 10 minutes after birth

• Continued need for resuscitation, including endotracheal or mask ventilation, at 10 minutes after birth

• Acidosis within 60 minutes of birth (defined as any occurrence of umbilical cord, arterial or capillary pH < 7.00)

• Base Deficit ≥ 16 mmol/L in umbilical cord or any blood sample (arterial, venous or capillary) within 60 minutes of birth

Infants that meet criteria A will be assessed for whether they meet the neurological abnormality entry criteria (B) by trained personnel:

**B. **Moderate to severe encephalopathy, consisting of altered state of consciousness (lethargy, stupor or coma) AND at least one of the following:

• hypotonia

• abnormal reflexes including oculomotor or pupillary abnormalities

• absent or weak suck

• clinical seizures

Infants that meet criteria A & B will be assessed by aEEG (read by trained personnel):

**C. **At least 30 minutes duration of aEEG recording that shows abnormal background aEEG activity or seizures. There must be one of the following:

• normal background with some seizure activity

• moderately abnormal activity

• suppressed activity

• continuous seizure activity

### Exclusion criteria

• Infants expected to be > 6 hours of age at the time of randomisation

• Major congenital abnormalities, such as diaphragmatic hernia requiring ventilation, or congenital abnormalities suggestive of chromosomal anomaly or other syndromes that include brain dysgenesis

Every effort will be made to ensure entry to the study before 3 hours of age. Delays should be avoided because there may be rapid attenuation of neuroprotection with delay in the start of cooling [69-71].

### Consent

Informed written consent will be obtained from a parent after a full verbal and written explanation of the study. The attending physician will meet with parents during the intervention period to ensure that they understand the study procedures and continue to consent to participate in the study.

Approval for the study has been obtained from the London Multicentre Research Ethics Committee and the Local Research Ethics Committee of each participating hospital.

### Randomisation

Patient allocation will be by central telephone randomisation, provided by the National Perinatal Epidemiology Unit (NPEU). As soon as parental consent has been obtained for an eligible infant, the recruiting TOBY clinician will telephone the randomisation service and obtain treatment assignment, which will be either to "intensive care with cooling" or "intensive care". Minimisation will be used to ensure balance between the groups with respect to severity of encephalopathy.

All infants recruited to the study will be cared for in a tertiary referral centre that has the equipment necessary for providing hypothermia treatment to the "intensive care with cooling" group ("Treatment Centres"). Infants may be recruited from hospitals that transfer babies to these centres. Babies that are thought to be eligible will be assessed by trained retrieval teams from the referral centres, who will perform the aEEG, seek consent if eligible, telephone the randomisation service and begin the cooling treatment, if allocated (see TOBY Handbook for more details).

### Clinical management

#### 1. Intensive care group

All infants who are randomised to this group will receive the present standard of clinical care. Infants will thus receive symptomatic therapy aimed at homeostasis. In particular, the management of the temperature of the infants will aim to maintain normothermia. All infants will be dried at birth, and kept warm during resuscitation. The infants will be cared for under an overhead radiant heater or in closed incubators, servo controlled to the infants' abdominal skin temperature, to maintain the rectal temperature at 37.0°C ± 0.2°C. Infants born in referring hospitals that are randomised to this group will be transferred to the Treatment Centre. The incubator heater will be adjusted during transport to maintain a rectal temperature as close to 37.0°C as possible.

#### 2. Intensive care with cooling group

All infants randomised to intensive care with cooling will be treated in closed incubators to facilitate cooling. Hypothermia will be maintained using the Tecotherm cooling system, which induces hypothermia by circulating fluid within a special mattress. A temperature thermostat can be regulated to alter the temperature of the fluid. Typically the thermostat should be set at 25–30°C to achieve a target rectal temperature of 33–34°C.

Infants born outside Treatment Centres who are allocated to intensive care with cooling will be nursed prior to transfer with the overhead radiant warmer turned off. During transport the infant will be nursed in a transport incubator. The incubator heater will be turned on and adjusted if necessary to maintain the rectal temperature between 33 and 34°C. Cooled gel packs will be placed around the infant if necessary to maintain the target temperature.

Some referring hospitals may have quick access to a Treatment Centre. If it is likely that the infant can be transferred within three hours of birth, assessment and randomisation may be postponed until admission to the Treatment Centre. These infants will receive the normal standard of care, including maintaining a rectal temperature of 37 ± 0.2°C, during the transfer

### Rewarming Procedures

When cooling is concluded 72 hours after randomisation, or earlier if clinical circumstances dictate, the rectal temperature will be allowed to rise by no more than 0.5°C per hour, to 37 ± 0.2°C. The thermostat set point of the Tecotherm system will be adjusted as needed, to rewarm the infants. The infants' temperature will be carefully monitored for at least 4 hours to prevent rebound hyperthermia, as this might be detrimental [71,72].

### Discontinuing cooling treatment

Cooling may be discontinued if any of the following occurs:

• Parents request that cooling be withdrawn prior to 72 hours after randomisation. This decision should be made in discussion with the TOBY Clinician whenever possible.

• Attending clinician withdraws cooling. Reasons will be recorded, and might include clinical, EEG and imaging evidence of severe, irreversible brain injury, or continued inability to maintain rectal temperature in the desired range.

• Baby receives extra-corporeal membrane oxygenation (ECMO).

If the parents or the attending paediatrician elect to discontinue cooling, then rewarming and follow-up procedures will be commenced. Apparent improvement on continuous aEEG recording, after trial entry, is not an indication for discontinuing cooling.

#### 3. Both groups

Treatment centres will provide a uniform standard of clinical care, to minimise potential bias that could arise from differential use of co-interventions. Following resuscitation, ventilatory requirement will be judged by assessing the infant's spontaneous breathing efforts and blood gas analysis. Infants who appear distressed will receive standard sedation. Seizures (whether noted on aEEG or clinically) will be treated using a standardised approach. Blood electrolyte analysis, urine volume and analysis and infant weight will be monitored to guide fluid management. See the TOBY Handbook for more details.

### Outcomes

#### Primary Outcome

• Combined incidence of mortality and severe neurodevelopmental disability in survivors at 18 months of age.

Neurodevelopment will be assessed, blind to study group, by a developmental paediatrician. Severe neurodevelopmental disability will be defined as the presence of at least one of:

• Bayley mental development scale (MDI) score < 70

• Gross motor function (GMF) neuromotor impairment Level 3–5

• Bilateral cortical visual impairment

#### Secondary Outcomes

Short term (before discharge from hospital):

1. Intracranial haemorrhage

2. Persistent hypotension

3. Pulmonary haemorrhage

4. Pulmonary hypertension

5. Prolonged blood coagulation time

6. Culture proven sepsis

7. Necrotising enterocolitis

8. Cardiac arrhythmia

9. Thrombocytopenia

10. Major venous thrombosis

11. Renal failure treated with dialysis

12. Pneumonia

13. Pulmonary air leak

14. Duration of hospitalisation

#### Long term (at 18 months)

1. Mortality

2. Severe neurodevelopmental disability

3. Multiple handicaps (defined as the presence of any two of the following in an infant; neuromotor disability (Level 3–5 on GMF classification), mental delay (Bayley MDI score < 70), epilepsy, cortical visual impairment, sensorineural hearing loss)

4. Bayley PDI score

5. Sensorineural hearing loss: ≥ 40 dB

6. Epilepsy (defined as recurrent seizures beyond the neonatal period, requiring anticonvulsant therapy at the time of assessment)

7. Microcephaly (head circumference more than 2 standard deviations below the mean).

### Data collection

Short-term outcomes (up to discharge from hospital) will be assessed from hospital notes. Major morbidity at 18 months will be assessed by a research paediatrician, blind to study allocation, using a standardised neurological examination and the revised Bayley Scales of Infant Development (BSID-II).

### Analysis

The relative risks of outcomes between the intensive care with cooling group and the intensive care group will be calculated, along with 95% confidence intervals.

Subgroup analyses will be carried out stratifying by:

(a) severity of encephalopathy at study entry (graded moderate or severe based on pre-specified aEEG criteria);

(b) interval from birth to randomisation (0–2 hours, 2–4 hours, 4–6 hours)

### Interim analyses

An independent Data Monitoring and Ethics Committee (DMEC) will be established, whose remit will be to review the study's progress. The DMEC will be independent of the trial organisers. Interim analyses will be supplied, in strict confidence, to the DMEC, as frequently as its Chair requests. Meetings of the committee will be arranged periodically, as considered appropriate by the Chair. In the light of interim data on the trial's outcomes, adverse event data, accumulating evidence from the NICHD study (see below) and any other relevant evidence (including updated overviews of the relevant randomised controlled trials), the DMEC will inform the Trial Steering Committee (TSC) if in their view (i) there is proof beyond reasonable doubt that the data indicate that any part of the protocol under investigation is either clearly indicated or contra-indicated, either for all infants or for a particular subgroup of trial participants or (ii) it is evident that no clear outcome will be obtained. Appropriate criteria for proof beyond reasonable doubt cannot be specified precisely. A difference of at least 3 standard deviations in the interim analysis of a major endpoint may be needed to justify halting, or modifying, such a study prematurely. If this criterion were to be adopted, it would have the practical advantage that the exact number of interim analyses would be of little importance, and so no fixed schedule is proposed. Unless modification or cessation of the study is recommended by the DMEC, the TSC, investigators, collaborators and administrative staff (except those who supply the confidential information) will remain ignorant of the results of the interim analysis. Collaborators and all others associated with the study, may write to the DMEC via the TOBY Co-ordinating Centre, to draw attention to any concern they may have about the possibility of harm arising from the treatment under study, or any other relevant matters.

The DMEC will liaise closely with a related trial of whole body cooling being conducted by the NICHD in the United States. This trial uses the same intervention but different eligibility criteria. Its results are likely to be relevant to decisions about continuation of recruitment to TOBY.

The membership of the DMEC is:

Professor Richard Cooke (Chair)

Dr Ann Johnson (Developmental Paediatrician)

Dr Sam Richmond (Neonataologist)

Dr Hugh Davies (Paediatrician and former MREC Chairman)

Dr Pat Yudkin (Statistician)

### Serious adverse event reporting

All unexpected serious adverse events will be reported to the TOBY co-ordinating centre at the National Perinatal Epidemiology Unit in Oxford within 24 hours. The study administrator will inform the Chair of the Data Monitoring and Ethics Committee and the Multicentre Research Ethics Committee of any serious adverse events.

### Sample size

The overall rate of the primary outcome in the non cooled group is assumed to be approximately 70%. A sample size of 188 infants will be necessary to detect a 30% relative risk reduction between the cooled and non cooled groups, at a significance level of 5% and with 80% power. The incidence of the primary outcome in the cooled group would be 49%. Allowing for a rate of loss to follow up of 10%, 207 infants will need to be recruited to reach this target.

However, this calculation needs to be modified to ensure that meaningful subgroup analyses can be performed for both infants with moderate and severe encephalopathy. The event rates of the primary outcome will be approximately 100% for infants with a severely abnormal aEEG, and about 60% for infants in the moderately abnormal group. Each subgroup needs to be large enough to detect a relative risk between the cooled and non cooled groups of about 0.6. For the moderately abnormal subgroup, the sample size required for 80% power will be 150.

It is expected that about 70% of infants will be in the moderately abnormal subgroup, so to ensure that 150 will be recruited to this subgroup, the total sample size will be 214. This would mean that 64 recruits would be in the severely abnormal subgroup, a sufficient number to detect the same difference in this subgroup with greater than 80% power. Allowing for 10% loss to follow up, the original total sample size to be recruited is 236.

## Discussion

Originally, it was the intention in the TOBY study to recruit a similar number of infants as the completed and other on-going studies (approximately 200 infants). Although there is an intention to continue further follow-up in the other studies, because of the difficulty of such follow-up, neither study will have recruited sufficient infants to enable assessment of the effect of cooling on outcome beyond 18 months of age. TOBY was in a unique position to ensure that a sufficient cohort of children is recruited to enable longer-term follow-up at age 6 years.

As the aim of whole body cooling in this group of babies is to provide neuroprotection, IQ is the most appropriate primary outcome of a long term follow-up study. IQ is standardised to 100 for "normal" children. However, in this group of children we can anticipate that IQ in either group is unlikely to approximate to the normal. In the small number of follow up studies that have assessed IQ in children with NE, the degree of later impairment is closely correlated with the degree of encephalopathy. For example, at age 8 years a group of 56 children born with mild encephalopathy had a mean IQ similar to their peer group (106 versus 112, using the Wechsler Intelligence Scales). In the 84 children with moderate encephalopathy the mean IQ was 95 (standard deviation 23) and for the 5 children with severe encephalopathy the mean IQ was 68 (standard deviation 21) [76].

One standard deviation below the mean for normal children is an IQ of 85. If we assume a mean of 85 in the non-cooled group of TOBY, with a standard deviation of 20, and wish to be able to detect half a "normal" standard deviation difference (i.e. 7.5), then this calculation will require 224 surviving children in total. The current mortality of babies recruited to TOBY is approximately thirty per cent. If this continues (and there are likely to be a small number of additional later deaths) and we assume an eighty per cent follow-up rate, then the required recruited sample size will need to be 400. However, relatively small changes in some of these assumptions, particularly the standard deviation of IQ, results in a wide variation in the number of survivors required to be able to detect the required difference.

This table of total trial sample sizes varies some of these assumptions to explore the effect on the sample size required:

### Table 2: Variations in mean IQ, standard deviations and mortality rates: their affect on sample size calculation

**Table 2:** Variations in mean IQ, standard deviations and mortality rates: their affect on sample size calculation.

						**Total trial size required assuming:**			
						
						**30% mortality**		**35% mortality**	
						
**Mean IQ in control group**	**SD**	**Mean IQ in cooled group**	**SD**	**Mean difference**	**Number required to detect this difference (80% power, 5% siq.)**	**80% follow up**	**75% follow up**	**80% follow up**	**75% follow up**
75	15	82.5	15	7.5	126	225	240	243	259
80	15	87.5	15	7.5					
85	15	92.5	15	7.5					
75	20	82.5	20	7.5	224	400	427	431	460
80	20	87.5	20	7.5					
85	20	92.5	20	7.5					
75	25	82.5	25	7.5	350	625	667	673	718
80	25	87.5	25	7.5					
85	25	92.5	25	7.5					

From these calculations, it appeared reasonable to suggest that the total trial sample size was increased from 236 infants to around 400 infants, to be sure that the trial had sufficient power to address important longer-term outcomes required to affect clinical practice. Otherwise it was likely that the trial would be underpowered to assess even modest differences (half a "normal" standard deviation) in IQ. Although the MRC declined to extend the study end date, because enrolment into TOBY exceeded expectation, 325 infants were finally enrolled into TOBY, which should still give a reasonable power to explore longer term outcomes.

### Organisation

#### Trial Steering Committee

The Trial Steering Committee (TSC) will provide overall supervision of the study on behalf of the Medical Research Council. Its terms of reference are:

1. To monitor and supervise the progress of the TOBY study towards its interim and overall objectives.

2. To review at regular intervals relevant information from other sources (e.g. related studies).

3. To consider the recommendations of the Data Monitoring and Ethics Committee.

4. In the light of 1, 2 and 3 above, to inform the MRC Council and relevant MRC Research Boards on the progress of the study.

5. To advise the MRC Council on publicity and the presentation of all aspects of the study.

The TSC will meet a least once per year. Membership of the TSC is:

Neil McIntosh (Chair; Neonatologist, Edinburgh)

Richard Parnell (SCOPE)

Harry Baumer (Neonatologist)

Caroline Doré (Statistician)

Diana Elbourne (Statistician)

Denis Azzopardi (Investigator)

Peter Brocklehurst (Investigator)

#### Project Management Group

The Project Management Group (PMG) will oversee the day-to-day running of the study, and will consist of the investigators and the trial staff, based at the TOBY Co-ordinating Centre (National Perinatal Epidemiology Unit, Oxford), and the clinical co-ordinating centre (Hammersmith Hospital, Imperial College London). The responsibilities of the PMG include:

i) recruitment of participating centres

ii) distribution and supply of data collection forms and other appropriate documentation for the trial

iii) data collection and management

iv) organisation of the follow-up

v) data entry and cleaning

vi) data analysis

vii) collection of adverse event data

viii) organising and servicing the Data Monitoring and Ethics Committee.

## Competing interests

The authors declare that they have no competing interests.

## Authors' contributions

The authors have all contributed to the design of the study protocol.

## Pre-publication history

The pre-publication history for this paper can be accessed here:


